# Electrochemical properties of La_0.6_Sr_0.4_CoO_3 − δ_ thin films investigated by complementary impedance spectroscopy and isotope exchange depth profiling^☆^

**DOI:** 10.1016/j.ssi.2013.12.016

**Published:** 2014-03-01

**Authors:** Markus Kubicek, Tobias M. Huber, Andreas Welzl, Alexander Penn, Ghislain M. Rupp, Johannes Bernardi, Michael Stöger-Pollach, Herbert Hutter, Jürgen Fleig

**Affiliations:** a Institute of Chemical Technologies and Analytics, Vienna University of Technology, Getreidemarkt 9, A-1060 Vienna, Austria; b University Service Center for Transmission Electron Microscopy, Vienna University of Technology, Wiedner Hauptstr. 8–10, A-1040 Vienna, Austria

**Keywords:** Mixed conductor, Impedance spectroscopy, Isotope exchange, Surface exchange, Diffusion, ToF-SIMS

## Abstract

The oxygen exchange and diffusion properties of La_0.6_Sr_0.4_CoO_3 − δ_ thin films on yttria stabilized zirconia were analyzed by impedance spectroscopy and ^18^O tracer experiments. The investigations were performed on the same thin film samples and at the same temperature (400 °C) in order to get complementary information by the two methods. Electrochemical impedance spectroscopy can reveal resistive and capacitive contributions of such systems, but an exact interpretation of the spectra of complex oxide electrodes is often difficult from impedance data alone. It is shown that additional isotope exchange depth profiling can significantly help interpreting impedance spectra by giving reliable information on the individual contribution and exact location of resistances (surface, electrode bulk, interface). The measurements also allowed quantitative comparison of electrode polarization resistances obtained by different methods.

## Highlights

• Complementary investigation by impedance spectroscopy and isotope exchange.
• Investigation of the same mixed conducting thin film at the same T with both methods.
• Quantitative comparison of resistive contributions.
• Discussion on interpretation of impedance data with different equivalent circuits.

## 1 Introduction

Mixed ionic and electronic conducting (MIEC) oxides are promising materials for electrochemical devices based on gas–solid interactions such as solid oxide fuel cells (SOFCs), gas sensors, or permeation membranes [1], [2], [3], [4]. Several analytical methods exist to investigate the catalytic activity of MIEC electrodes towards the oxygen reduction reaction (ORR) with two of the most important approaches being electrochemical impedance spectroscopy (EIS) and ^18^O isotope exchange depth profiling (IEDP). EIS yields information on resistive and capacitive contributions of MIEC electrodes on ionic conducting substrates. Many properties such as the catalytic activity of surfaces, oxygen non-stoichiometry, chemical diffusion, conductivities, transport reactions across solid|solid phase boundaries, or the formation of impurity phases can thus be indirectly probed. However, the correct interpretation of impedance spectra is crucial for the validity of the extracted parameters. This interpretation can be trivial for simple spectra [5], [6] but in complex systems impedance analysis is often very difficult and far from being unambiguous. An equivalent circuit for mixed conductors was introduced in Ref. [7], but it is also restricted in its applicability, cf. Ref. [8]. Compared to EIS, oxygen isotope exchange and subsequent depth profiling [9] has the simpler methodology for data interpretation even though the experiment itself is more elaborate. There, the properties of oxygen exchange are tested by providing an isotopic tracer (e.g. ^18^O) via the gas phase and establishing a time dependent concentration depth profile in a sample. The local tracer concentration is determined by secondary ion mass spectrometry (SIMS) and from the resulting depth profile, ion exchange and diffusion related parameters can be extracted.

While impedance spectroscopy is a quite common method to investigate mixed conducting thin film electrodes, [6], [10], [11], [12] oxygen tracer experiments are often performed on bulk samples [13], [14], [15], [16]. Recently, several IEDP measurements of mixed conducting cathode materials were published with the oxide films being deposited on insulating substrates [17], [18], [19]. However, to the best of the authors' knowledge no study so far reported experiments with both techniques being applied on the same films at the same temperature. This contribution reports the results of a study applying EIS and IEDP to one and the same La_0.6_Sr_0.4_CoO_3 − δ_ (LSC) thin film in order to get complementary results on the resistive contributions of the oxygen reduction kinetics on such films. As electrical measurements require an oxygen ion conductor, yttria stabilized zirconia (YSZ) was used as substrate for LSC films with two different grain sizes. Quantitative material parameters are deduced from both types of experiments and comparison of the data allowed testing the appropriateness of analysis models.

## 2 Experimental

LSC powder was prepared via the nitrate/citrate (Pechini) route. The following high purity base materials were used: Co 99.995%, SrCO_3_ 99.995%, La_2_O_3_ 99.999%, HNO_3_ 70% in H_2_O, 99.999% purity, citric acid monohydrate 99.9998% (all Sigma–Aldrich). Pulsed laser deposition (PLD) target was produced by isostatic pressing (5.2 kbar, 2 min) and sintering (1150 °C, 12 h). Dense La_0.6_Sr_0.4_CoO_3 − δ_ thin films with 200 nm thickness were prepared on 10 × 10 × 0.5 mm^3^ YSZ (100) single crystals by pulsed laser deposition (PLD). A target to substrate distance of 7.0 cm was chosen and the depositions were performed under 0.04 mbar O_2_, at 400 mJ/pulse laser energy, 10 Hz pulse frequency, and 27 min deposition time. Two different substrate temperatures during film growth, measured with a pyrometer (Heitronics KT-19.99), were used to prepare thin films with different grain size denoted LSC-LT (450 °C) and LSC-HT (600 °C). Phase purity of the PLD target was investigated by X-ray diffraction (XRD) in Bragg–Brentano geometry (X'Pert PRO diffractometer PW 3050/60, PANalytical). For thin films, XRD measurements were performed in parallel beam geometry on a D8-Discover instrument (Bruker AXS) which was equipped with a General Area Detection Diffraction System (GADDS). Information along the theta axis and the chi axis (tilted grains) could be obtained at the same time. Nanostructure, and grain sizes of both types of thin films were investigated by transmission electron microscopy (TEM) imaging of thin film cross-sections (FEI TECNAI F20).

Before isotope exchange and electrical measurements, LSC thin films were microstructured by photolithography in order to prepare circular microelectrodes with 200 μm diameter. Oxygen isotope exchange experiments were performed by heating to 400 °C in air (12 K/min), changing the atmosphere to 200 mbar 97.1% ^18^O isotope enriched oxygen (Campro Scientific) for 5 min, and then cooling to room temperature with 60 K/min to freeze the tracer diffusion profile. A pre-annealing in oxygen as often reported in literature was avoided for two reasons. One reason is that, due to the ^18^O concentrations above the natural abundance in bottled oxygen [20] a tracer profile would already be created in the sample before the actual tracer exchange experiment. The even more important reason is that due to the short diffusion times of ~ 5 min an experimental procedure of: pre-annealing – cooling – gas exchange – heating – annealing would cause a significant contribution to the tracer profiles from the heating step. Gas exchange with evacuation at annealing temperature (the procedure used) annihilates any pre-annealing effect due to the very fast chemical diffusion in LSC (few s) at the annealing temperatures. By the procedure used, a tracer profile containing chemical and tracer incorporation/diffusion is created. However for the used experimental setup, the influence of chemical tracer incorporation in the experiments is negligibly small due to the orders of magnitude higher amount of ^18^O incorporated by tracer exchange as also explained in more detail in Ref. [17]. The resulting isotope diffusion profiles were subsequently measured by depth profiling with ToF-SIMS (TOF.SIMS 5, ION-TOF). 25 kV Bi^+^ primary ions were used in CBA measurement mode.[21] Negative secondary ions were analyzed in areas of 100 × 100 μm^2^ using a raster of 512 × 512 measured points. For depth-profiling, 2 kV Cs^+^ ions (500 × 500 μm^2^, ca. 105 nA) were used for sequential ablation of the surface between measuring mass spectra. For charge compensation, a low energy electron flood gun (10 V) was employed.

Following the SIMS measurements, the same thin films (though different microelectrodes) were investigated by impedance spectroscopy. Here, 200 μm microelectrodes were contacted by gold covered steel needles (EGON 0.4, Pierenkemper) and measured with an Alpha-A high performance frequency analyzer (Novocontrol, Germany) versus a macroscopic LSC counter electrode at 400 °C in air. AC frequencies of 1 MHz to 0.07 Hz with 10 mV effective amplitude were applied to obtain impedance spectra. More details on such microelectrode measurements can be found in Ref. [11].

## 3 Results

### 3.1 Thin film characterization

Thin films were investigated by XRD using theta-2theta and chi-scans. For both LSC-LT and LSC-HT no impurity phases were found and all reflexes could be attributed to pseudo-cubic perovskite phase. Lattice parameters of the thin films were analyzed from the maxima position of 4 strong reflexes each and the values of a = 0.3826 ± 0.0006 nm for LSC-LT and a = 0.3830 ± 0.0003 nm for LSC-HT were calculated.

**Fig. 1:**
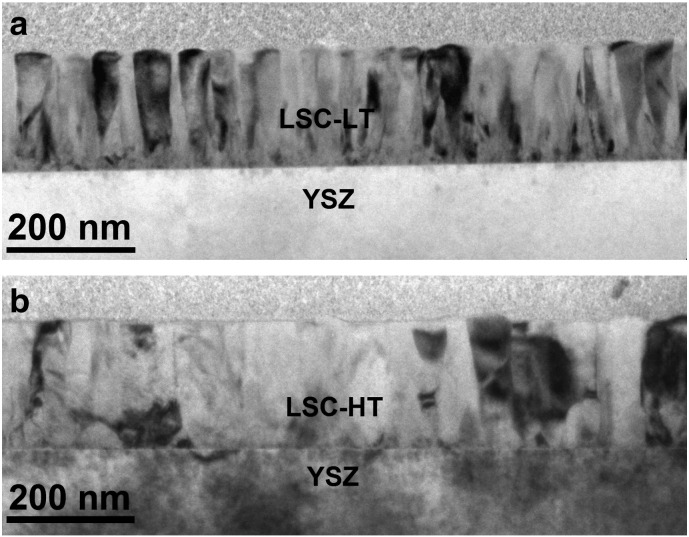
TEM thin film cross sections showing columnar growth of 200 nm La_0.6_Sr_0.4_CoO_3 − δ_ PLD thin films. Differences in grain size and microstructure are visible for LSC-LT (a) and LSC-HT (b).

**Fig. 2:**
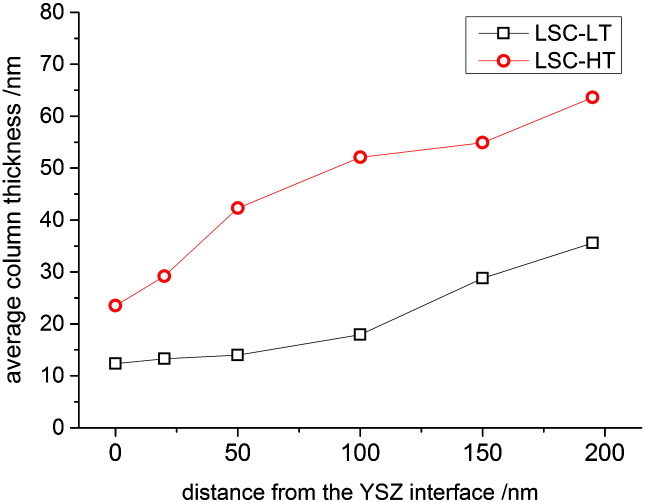
Average column thickness for LSC-LT and LSC-HT evaluated for different distances from the LSC|YSZ interface from more than 12 μm TEM cross section length each.

TEM cross section imaging of the two film types as shown in Fig. 1 was performed in order to analyze their nanostructure and grain size. Both LSC film types are dense and columnar growth is observed which is expected for PLD grown films on YSZ with the parameters as used.[22] Differences between the two film types are a result of the different deposition temperature, and the obvious difference in grain size was investigated in more detail. About 20 cross section images, similar to those shown in Fig. 1 were recorded, and the number of grain boundaries was analyzed in different depths of the film. Thus, the average cross-section column thickness was calculated from a representative total cross section length of more than 12 μm each for LSC-LT and LSC-HT films as shown in Fig. 2. LSC-HT has the larger grain size throughout the whole film thickness compared to LSC-LT. The largest average distance between grain boundaries is present close to the surface with ~ 64 nm for LSC-HT and ~ 33 nm for LSC-LT. Close to the LSC|YSZ interface both thin films have the highest grain boundary density and the smallest grains with an average distance of ~ 24 nm (LSC-HT) and ~ 13 nm (LSC-LT).

### 3.2 Electrochemical impedance spectroscopy

**Fig. 3:**
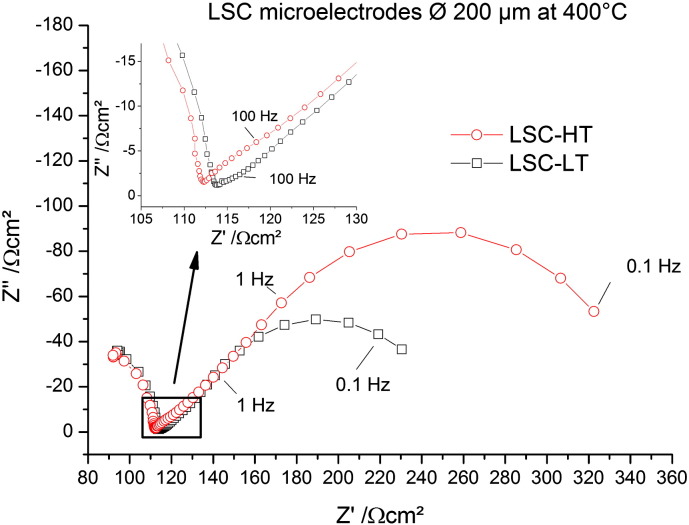
Nyquist plot for EIS measurements on LSC microelectrodes on YSZ. LSC-LT has the lower total electrode resistance. The medium frequency part exhibiting differences between the two spectra is shown as inset.

In Fig. 3 impedance spectra are shown, measured on circular 200 μm microelectrodes of the differently prepared LSC thin films. Several features are visible in both spectra. At the highest frequencies a part of a semicircle is visible which can be attributed to the ionic spreading resistance of YSZ underneath the microelectrode. The resistance depends on the size of the microelectrode and is inversely proportional to the ionic conductivity of YSZ. From this relation and reference measurements of the temperature dependent ionic conductivity of YSZ single crystals it is possible to calculate an average temperature of the microelectrode as discussed in Ref. [11]. In our measurements the set temperature of the furnace was chosen such that the YSZ spreading resistance corresponded to a temperature of 400 ± 1 °C.

At lower frequencies the impedance contributions of the electrode become visible. A Warburg-like shape of the electrode contribution with almost a 45° straight line is found at medium frequencies, followed by a semicircle-like part at low frequencies. Even though these general features are similar for LSC-LT and LSC-HT, also some differences can be observed between their impedance spectra. Most obvious, the total electrode resistance is smaller for LSC-LT. Differences of the shape are visible in the intermediate frequency part shown in the inset in Fig. 3. LSC-HT approaches the 45° range from larger phase angles (semicircle like) while LSC-LT exhibits a very flat spectrum part above ca. 100 Hz. Impedance contributions in this intermediate frequency range are often attributed to the electrode|electrolyte interface.[6]

### 3.3 Isotope exchange depth profiling

**Fig. 4:**
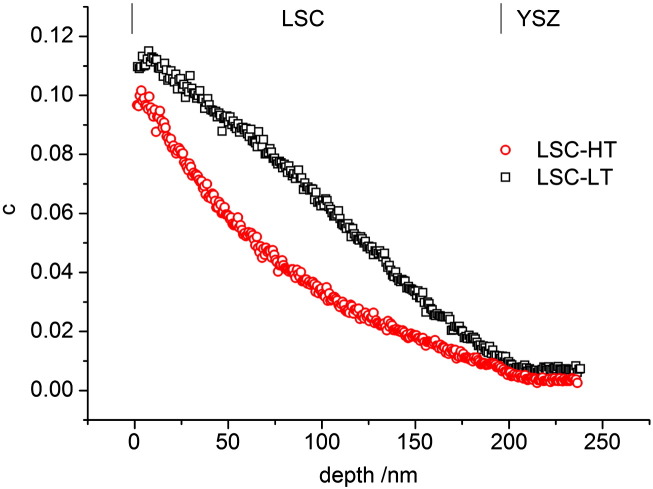
Oxygen isotope depth profiles measured on the same samples as in Fig. 3 after annealing at 400 °C for 5 min. A different amount of ^18^O incorporated into YSZ, a different bending of the ^18^O concentration curves of LSC-LT and LSC-HT, and a different drop at the interface are discernible.

In Fig. 4 the oxygen isotope depth profiles are shown, measured by ToF-SIMS on the same samples after annealing in ^18^O enriched atmosphere at 400 °C for 5 min. The isotope fractions c are calculated according to

(1) c=countsO18countsO16+countsO18.

Both depth profiles exhibit a surface isotope fraction of about 10% followed by a drop in concentration in the LSC thin film which can be attributed to the limited ionic conductivity of LSC. Then, at 200 nm depth, a transition to a very flat profile in YSZ follows which reflects the high ionic conductivity there. Several differences can be observed between the profiles. The total amount of incorporated ^18^O is higher in LSC-LT, the slope of the concentration decay in LSC is different, and a small step in concentration at the interface is observed for LSC-HT and not for LSC-LT. The general tendency of LSC-LT having the lower polarization resistance in EIS and showing the higher exchange rate of tracer is therefore matching well, but for an exact analysis and comparison of oxygen transport parameters and resistance contributions, fitting methods are necessary to analyze both sets of data.

## 4 Data analysis

### 4.1 Impedance spectroscopy

**Fig. 5:**
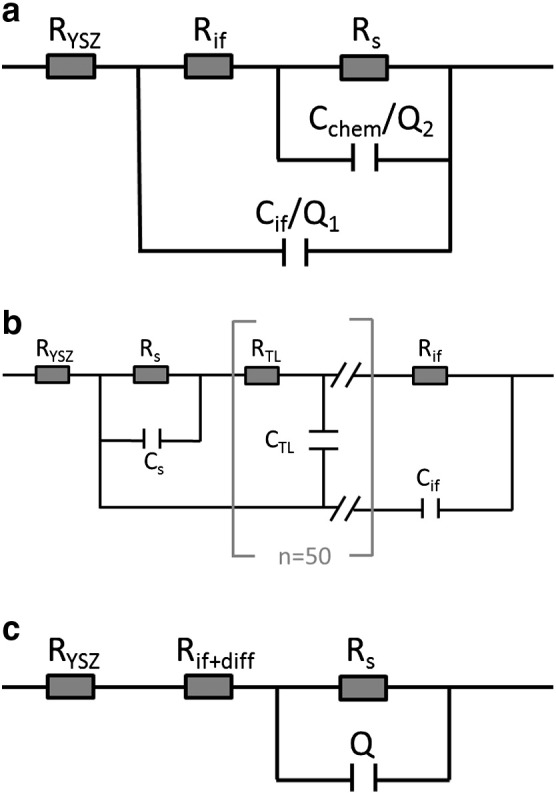
Equivalent circuits used for fitting of impedance spectra. The circuits shown in (a) and (b) are adapted from circuits derived in Ref. [7]; high electronic conductivity is assumed and the transmission line is approximated by 50 R_TL_, C_TL_-elements (R_diff_ = 50R_TL_ and C_chem_ = 50C_TL_). Circuit (a) results when neglecting R_TL_ (i.e. ion transport resistances) and C_s_ in (b). The incomplete equivalent circuit shown in (c) was used to fit only the low frequency impedance using a beforehand determined and fixed value of R_YSZ_.

Generalized equivalent circuits for mass and charge transport in mixed conducting systems are extensively discussed by Jamnik and Maier.[7] The equivalent circuits shown in Figs. 5a,b are slightly adapted from Ref. [7]. The transmission line model in Fig. 5b represents a mixed conducting electrode with surface related resistance R_s_, ionic transport resistance R_diff_ (here a sum of 50 equivalent resistances R_TL_ in a transmission line) and interfacial resistance R_if_. A reduced model for surface controlled oxygen exchange and negligible R_diff_ is shown in Fig. 5a. The capacitors, C_s_, C_if_ and C_diff_ = 50 C_TL_ represent the capacitances of surface, interface, and the chemical bulk capacitance (in a transmission line), respectively. Q is a constant phase element with the impedance:

(2) $$Z_{Q}=\frac{1}{{\left(\mathrm{i\omega}\right)}^{n}P}.$$

For *n* = 1, Q is equivalent to a capacitor with capacitance P. By using Q instead of C in RC elements (sometimes called “Cole Element”) it is possible to model slightly depressed semicircles, which are regularly observed due to non-idealities in samples.

The model shown in Fig. 5c is an incomplete equivalent circuit which was used to fit only the low frequency part of the impedance spectra. In this case, the spreading resistance of the electrolyte R_YSZ_ was determined beforehand from the axis intercept of the high frequency arc and fixed. Then only low frequency points, i.e. the main part of the large semicircle, were used for fitting (LSC-HT: 8 Points 1–0.1 Hz, LSC-LT: 7 Points 0.6–0.07 Hz. This arc was attributed to the electrode surface reaction in accordance with studies demonstrating a clear correlation of low frequency arc and surface oxygen exchange.[5], [23] The additional resistance value of the electrode R_if+diff_ includes all other contributions to the total electrode resistance (e.g. R_if_, R_diff_).

**Fig. 6:**
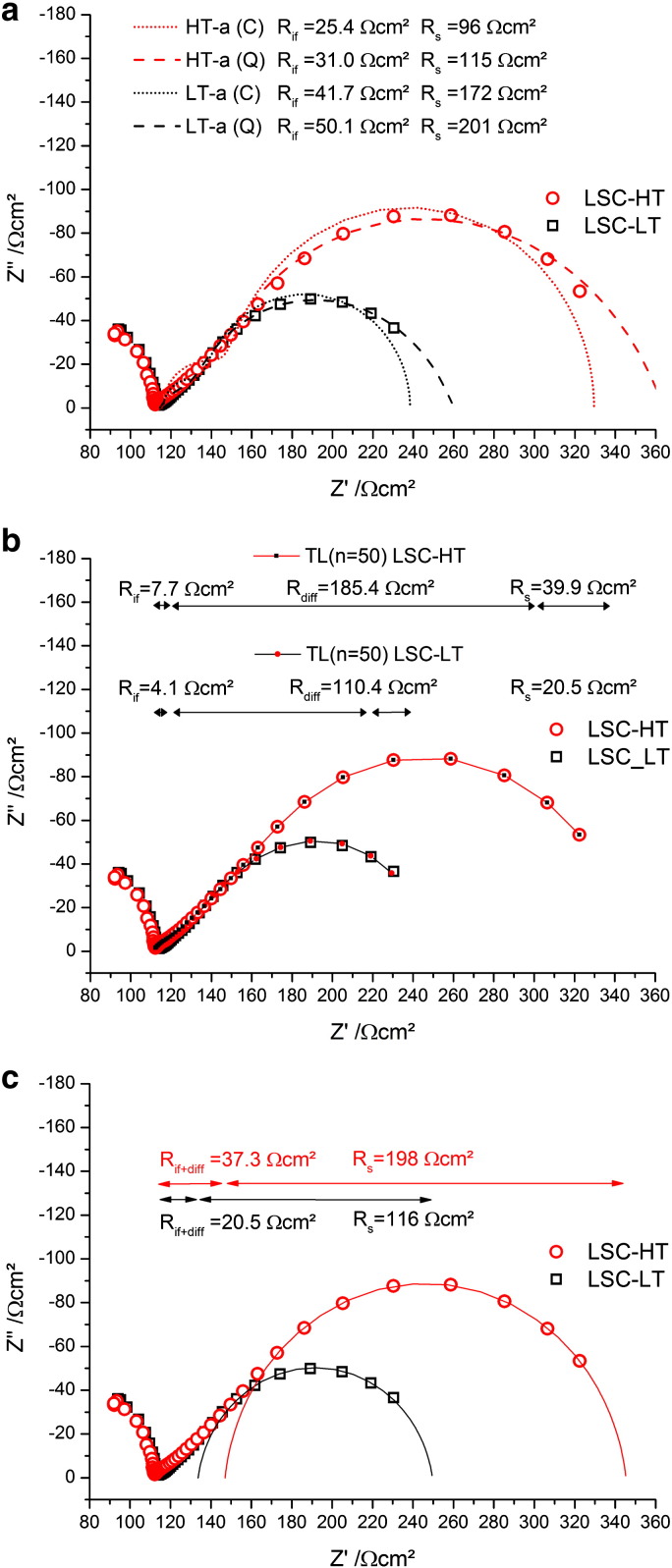
Fitting curves for the impedance spectra generated with the different equivalent circuits. For the fits in (a),(b),(c) the corresponding models in Figs. 5a,b,c were used.

The results of fitting the impedance spectra with these different equivalent circuits are shown in Figs. 6a,b,c. The fits using the reduced circuit (Fig. 5a) could often be applied in literature [24] to quantify spectra of similar electrode materials at higher temperature. Here, however, the corresponding model is not suited well to fit the impedance spectra, see Fig. 5a. The fits give a larger resistance R_s_ for the electrode surface and a smaller resistance R_if_ for the LSC|YSZ interface, but neither the high frequency part nor the low frequency part is well reproduced even though constant phase elements are used (a (Q)). As in this model the oxygen diffusion resistance in LSC is neglected, the failure to reproduce the spectra gives evidence that diffusion (also indicated by the 45° angle in Nyquist plot) may indeed contribute significantly to the total electrode resistance.

Fitting with the equivalent circuit in Fig. 5b, which includes ion diffusion in a transmission line of 50 identical elements, reproduces the measured impedance spectra very well (cf. Fig. 6b). The fitting parameters of this model suggest a very small interface resistance and a small surface resistance. The major part of the electrode resistance is there explained by the resistances in the transmission line which depend on the ionic conductivity of LSC. This distribution of resistances would suggest that the rate limiting step of the overall oxygen reduction in these LSC films is chemical bulk diffusion.

By using the fitting model with only one RQ element in Fig. 5c, the low frequency part of the impedance spectrum is also well reproduced with a single slightly depressed semicircle (*n* ~ 0.96). Assigning this resistance to the electrode surface leaves only a minor part of the total electrode resistance to the contributions of diffusion and the LSC|YSZ interface. In this model, the surface exchange of oxygen would be rate limiting, in contrast to the interpretation derived from the fit in Fig. 6b. This discussion reveals that an unambiguous conclusion what is the rate limiting step is hardly possible based on these impedance data alone.

### 4.2 Isotope exchange depth profiling

**Fig. 7:**
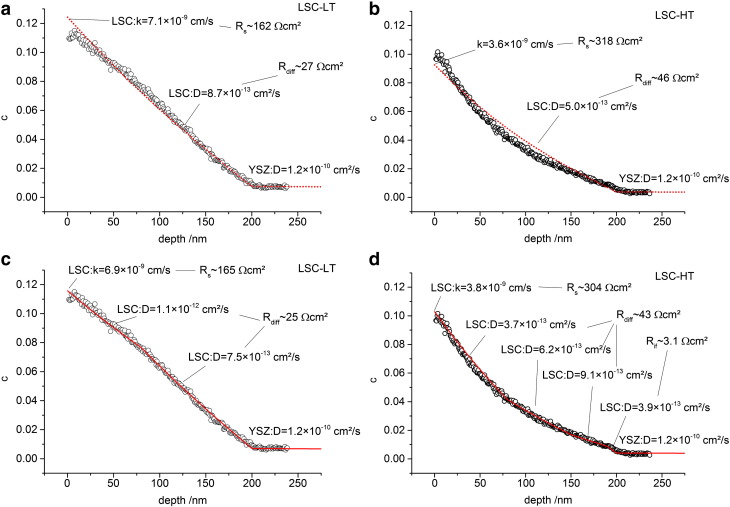
Fitting curves for the tracer depth profiles using only one diffusion coefficient for LSC (a,b) and using variable diffusion coefficients (c,d) for LSC-LT (a,c) and LSC-HT (b,d). Calculated individual resistance contributions are also shown.

Owing to the diffusion in two phases (LSC, YSZ) an analytical solution for analyzing the measured tracer profiles is not available. COMSOL finite elements software was therefore used to numerically solve the diffusion problem with boundary conditions *c* = 0.971 in the gas atmosphere (^18^O concentration in the gas) and *c* = 0.00205 (natural abundance of ^18^O) in the LSC layer as well as in YSZ before tracer diffusion takes place. Two different models were used to numerically analyze the oxygen isotope depth profiles in Fig. 4. The simpler model consists of three parameters, k*, D*_LSC_ and D*_YSZ_. The value of D*_YSZ_ was fixed at 1.2 × 10^− 10^ cm^2^/s in accordance with conductivity measurements, and only k* and D*_LSC_ were varied to fit the measured depth profiles. This model could not exactly reproduce the experimental results (see Figs. 7a,b). Particularly the profile in LSC-HT shows significant deviations from the fit result. Spatially varying diffusion coefficients in LSC were therefore allowed in a second model. For LSC-HT this included three larger zones in LSC with different D* values and a short zone (10 nm) directly at the LSC|YSZ interface. The latter represents an interface resistance. The LSC-LT films were analyzed in terms of two zones of different D* without an interface region due to absence of a sharp drop there (cf. Fig. 4). With this second model better matching fits of the isotope depth profiles were achieved and parameters for surface exchange and diffusion could be extracted, see Figs. 7c,d.

From the fit parameters of both models it is possible to calculate resistive contributions by using the Nernst–Einstein relation. In Eq. 3, this is exemplarily shown for the surface exchange coefficient [6], [25], [26].

(3) $$R_{s}=\frac{k_{B}T}{4e^{2}k^{q}c_{0}}$$

Here, k^q^ is the electrical surface exchange coefficient, k_B_ is Boltzmann's constant, T the temperature, e the elementary charge and c_0_ the total concentration of lattice oxygen (8.90 × 10^− 2^ mol/cm^3^ LSC-LT, 8.87 × 10^− 2^ mol/cm^3^ LSC-HT) calculated from X-ray diffraction data of the La_0.6_Sr_0.4_CoO_3 − δ_ thin films and neglecting oxygen non-stoichiometry. An error in the range of ~ 1% (δ ~ 0.03 [27], [28], [29]) can be expected which is not large compared to the necessary simplifications and experimental errors. An analogous calculation is possible for the diffusion parameters using D^q^/thickness instead of k^q^. As for this calculation the electrical parameters k^q^ and D^q^ are required, and in the isotope exchange experiment only the tracer parameters k* and D* can be determined, a correction factor is necessary to consider their different values [25] (Eq. 4). For the diffusion coefficients this factor is the Haven ratio H and we therefore obtain

(4) $$D^{q}\cdot H=D⁎.$$

In perovskite-type oxides H is often assumed to be very close to the correlation factor of 0.69 [30]; this value is also used in the following. The correlation factor of surface exchange (f_s_) depends on the exact elementary mechanism. As a first approximation the same ratio as for diffusion was used according to

(5) $$k^{q}\cdot f_{s}=k⁎,f_{s}\approx H.$$

Even though the quality of the fit is better when using different diffusion coefficients, the extracted values of the resistive contributions are very similar for the two models. In both LSC thin films the largest resistive contribution can be attributed to the electrode surface (~ 160 Ωcm^2^ and ~ 310 Ωcm^2^ for LSC-LT and LSC-HT, respectively). This dominance of the surface resistance is also in accordance with literature results for LSC investigated by impedance spectroscopy at higher temperatures.[24] Transforming the interfacial concentration drop in LSC-HT into an LSC|YSZ interface resistance shows that it amounts only to a very small fraction of the total electrode resistance (~ 3 Ωcm^2^). Larger than this interface resistance, but still smaller than the dominating surface resistance, is the ionic diffusion resistance of LSC. Interestingly, significant changes of the diffusion coefficient with depth were extracted for LSC-HT. Here, closer to the interface more than a factor of 2 faster diffusion than close to the surface was found. For LSC-LT only slight inhomogeneities of the diffusion coefficient were obtained, and here diffusion was faster closer to the surface. The reasons for these inhomogeneities as well as for the differences between the LSC-LT and LSC-HT films are most probably caused by the nanostructure. A possible difference of oxide ion conduction in LSC grains and grain boundaries may play a role here which leads to changes in depth due to the changing grain boundary density as shown in Fig. 2. The increased average diffusion coefficient closer to the interface as observed for LSC-HT would suggest faster diffusion of oxide ions in or along grain boundaries than in the bulk. There is some arbitrarity in defining number and size of regions with different diffusion coefficients in Fig. 7, but essential in our context are primarily the total resistances of diffusion and the fact that measurable inhomogeneities exist at all.

### 4.3 Comparison of the extracted parameters from EIS and IEDP

In Table 1, Table 2 the resistive parameters extracted with the different measurement and fitting methods are compared for LSC-LT and LSC-HT.

When comparing the total electrode resistance R_LSCtotal_, a systematic difference can be noted between the values from impedance measurements and isotope exchange, showing higher resistance values in the tracer studies. The difference of about 30–50% can have several causes. The true correction factor between k* and k^q^ is unknown and the chosen value f_s_ = 0.69 for k can be the reason for discrepancies between the resistances obtained by the different methods. For a lower factor f_s_ = 0.5 in Eq. 5 the total resistances would fit very well to the values determined with EIS. Another possible source for a systematic deviation is the temperature. In microelectrode measurements a temperature gradient in the sample is caused by the contact tip. Even if the temperature is corrected by the YSZ spreading resistance, a temperature distribution over the electrode area is present that can affect the effective electrode temperature.[31] For typical temperature dependencies of LSC electrode resistances (E_a_ 1.3–1.6 eV [15], [24]) a 30% change of the total resistance is already caused by a temperature difference of less than 10 °C. Accordingly, the still rather similar resistance values obtained by the two techniques are regarded as indication that indeed both methods probe the same electrochemical processes and are appropriate for analyzing the oxygen reduction kinetics of mixed conducting electrodes.

Fit results of the impedance data show that the total resistances of the EIS models with the transmission line (Fig. 6b) and with only one RQ element (Fig. 6c) are matching well, but the distribution of the resistances to the individual processes is very different. Comparing this to the data extracted from ^18^O experiments we find the simple model c, fitting only one semicircle for the surface resistance, is matching much better. This becomes obvious from the ratio of the surface resistance to the other electrode resistances shown in the last row of Tables 1,2. Here model c (Fig. 5c) yields values of 5–6, matching best to the values of 6–7 found in tracer experiments. The model including the full transmission line finds completely different ratios of about 0.2 here. One might get the impression that the model with the transmission line is simply over-parameterized and it should be possible to shift the predominant resistance from R_diff_ to R_s_ while remaining a good fit quality. This assumption was investigated by fixing the surface resistance to higher values up to the resistance found by model c. However, in these cases the quality of the fit was much lower, and performing a linear least square fit with such starting parameters again yielded fit results with dominating R_diff_. From such a quantitative comparison it is also concluded that the more semicircle-like intermediate frequency part of LSC-HT (Fig. 3) can be attributed to existence of a small interfacial resistance R_if_ of LSC-HT and finds its counterpart in the concentration step of the tracer profile (cf. Fig. 7d). Concentration step as well as semicircle-like feature at these frequencies are absent for LSC-LT.

This discussion shows that the apparently exact model b fails to correctly analyze the impedance data. It should be kept in mind however, that also model b is based on assumptions, e.g. spatially homogeneous R_TL_ values (constant ionic conductivity) while the profiles of the tracer diffusion experiments strongly suggest that this is not fulfilled here. Further, also the chemical capacitance could accordingly vary strongly with depth. Both of these inhomogeneities with depth can result from the nanostructure of PLD grown LSC thin films and the change of grain size and grain boundary density with depth as shown in Fig. 2. This shortcoming of model b could lead to the wrong material parameters resulting from the fit procedure. Accordingly, a misinterpretation of the oxygen reduction kinetics and identification of an erroneous rate limiting step may easily happen when only relying on impedance data. Combining two independent and complementary measurement methods in order to investigate the electrochemical properties of mixed conducting electrodes is clearly advantageous.

## 5 Conclusions

Impedance spectroscopy and isotope exchange depth profiling were used to investigate the oxygen exchange and transport properties of LSC thin film electrodes on YSZ single crystals. Two types of LSC thin films (LSC-LT and LSC-HT, prepared at different temperatures) were considered. The same films were consecutively analyzed by the two methods yielding complementary information. The choice of the correct equivalent circuit for fitting of the impedance spectra and for separating the total electrode resistance into its different contributions proved to be intricate. Different fit models suggested different processes to be rate limiting (surface exchange, diffusion). However, in oxygen tracer experiments, it could be unambiguously shown that the oxygen exchange at the surface is rate limiting and the best suited model for impedance analysis could thus be identified. Data analysis also allowed a quantitative comparison of the resistances of the electrode surface, of diffusion in the electrode and of oxygen transport across the LSC|YSZ interface extracted by impedance and tracer measurements. Only rather small differences of the calculated total electrode resistances were observed between the two methods. Further, relative importance of surface exchange and diffusion correspond well in both types of experiments and a small impedance contribution of the LSC|YSZ interface resistance found only for LSC-HT in tracer experiments is well matching to differences in the impedance response of LSC-LT and LSC-HT. This shows that analysis of data from impedance spectroscopy can be significantly improved by complementary IEDP experiments especially when spatial inhomogeneities such as different grain sizes with depth are present. IEDP gives reliable information on the localization of resistances (surface, electrode bulk, interface) or inhomogeneities of diffusion in depth.

## Figures and Tables

**Table 1:** Resistive contributions of the LSC-LT thin film electrodes extracted from EIS and IEDP measurements. Models a–c correspond to the equivalent circuit models shown in Fig. 5. (C), (Q) indicate whether capacitances or constant phase elements were used. ^18^O 1D* and ^18^O var. D* correspond to the fits of tracer depth profiles using a single or variable diffusion coefficients in LSC. R_LSCtotal_ is the sum of all resistances attributed to the LSC electrode.

LSC-LT	R_if_/Ωcm^2^	R_diff_/Ωcm^2^	R_s_/Ωcm^2^	R_LSCtotal_/Ωcm^2^	R_s_/(R_if_ + R_diff_)
Model a (C)	25.4	–	96.7	122	3.8
Model a (Q)	31.0	–	114.6	146	3.7
Model b	4.1	110.4	20.5	135	0.18
Model c	20.5		116	137	5.7
^18^O 1D*	–	27	162	189	6.0
^18^O var. D*	–	25	165	190	6.6

**Table 2:** Resistive contributions of the LSC-HT thin film electrodes extracted from EIS and IEDP measurements with analogous abbreviations as in Table 1.

LSC-HT	R_if_/Ωcm^2^	R_diff_/Ωcm^2^	R_s_/Ωcm^2^	R_LSCtotal_/Ωcm^2^	R_s_/(R_if_ + R_diff_)
Model a (C)	41.7	–	172	214	4.1
Model a (Q)	50.1	–	201	251	4.0
Model b	7.7	185.4	39.9	233	0.21
Model c	37.3		198	235	5.3
^18^O 1D*	–	46	318	364	6.9
^18^O var. D*	3.1	43	304	350	6.6
